# SOX2-Sensing: Insights into the Role of SOX2 in the Generation of Sensory Cell Types in Vertebrates

**DOI:** 10.3390/ijms24087637

**Published:** 2023-04-21

**Authors:** Sara Mercurio

**Affiliations:** Department of Biotechnologies and Biosciences, University of Milan-Bicocca, Piazza della Scienza 2, 20126 Milan, Italy; sara.mercurio@unimib.it

**Keywords:** SOX2, sensory, transcription factor, taste, smell, hear, touch

## Abstract

The SOX2 transcription factor is a key regulator of nervous system development, and its mutation in humans leads to a rare disease characterized by severe eye defects, cognitive defects, hearing defects, abnormalities of the CNS and motor control problems. SOX2 has an essential role in neural stem cell maintenance in specific regions of the brain, and it is one of the master genes required for the generation of induced pluripotent stem cells. *Sox2* is expressed in sensory organs, and this review will illustrate how it regulates the differentiation of sensory cell types required for hearing, touching, tasting and smelling in vertebrates and, in particular, in mice.

## 1. Introduction

There are five main senses that we use to interact with the world around us, that is, sight, hearing, taste, smell and touch. Each one of them receives inputs from the environment by means of an organ with specialized structures that have receptor cells for specific stimuli. Sensory pathways include neurons that link the receptors at the periphery with the CNS regions that process sensory information. A certain organ within the body corresponds to each of these senses: sight is mediated by the eyes; hearing, by the ears; taste, by the mouth; smell, by the nose; touch, by the skin.

The SOX2 transcription factor is expressed in the nervous system from when it starts to form [1]. It is required for neural stem cell self-renewal in the forebrain, in particular in the hippocampus, the part of the brain important for memory formation, in which neurogenesis continues into adulthood [2,3,4,5,6]. Recently, it was shown to be also required in differentiated cell types that include projection neurons and specialized glial cells, such as Müller glia in the retina and Bergmann glia in the cerebellum [7,8,9,10,11].

*Sox2* mutation in humans leads to a rare disease characterized by anophthalmia or microphthalmia, hippocampus hypoplasia, seizures, motor control problems, cognitive defects and, sometimes, hearing loss [12,13,14]. *Sox2* expression is highly conserved between humans and mice; indeed, mouse models have been instrumental in reproducing and studying some of the human phenotypes [4,15].

This review will illustrate some recent studies on the role of SOX2 in the formation of sensory organs and, in particular, in the generation of the cell types required to sense the environment around us.

The role of SOX2 in the visual system will not be discussed, since it has been previously described [3,16,17].

## 2. TOUCH: SOX2 and Merkel Cells

**Figure 1:**
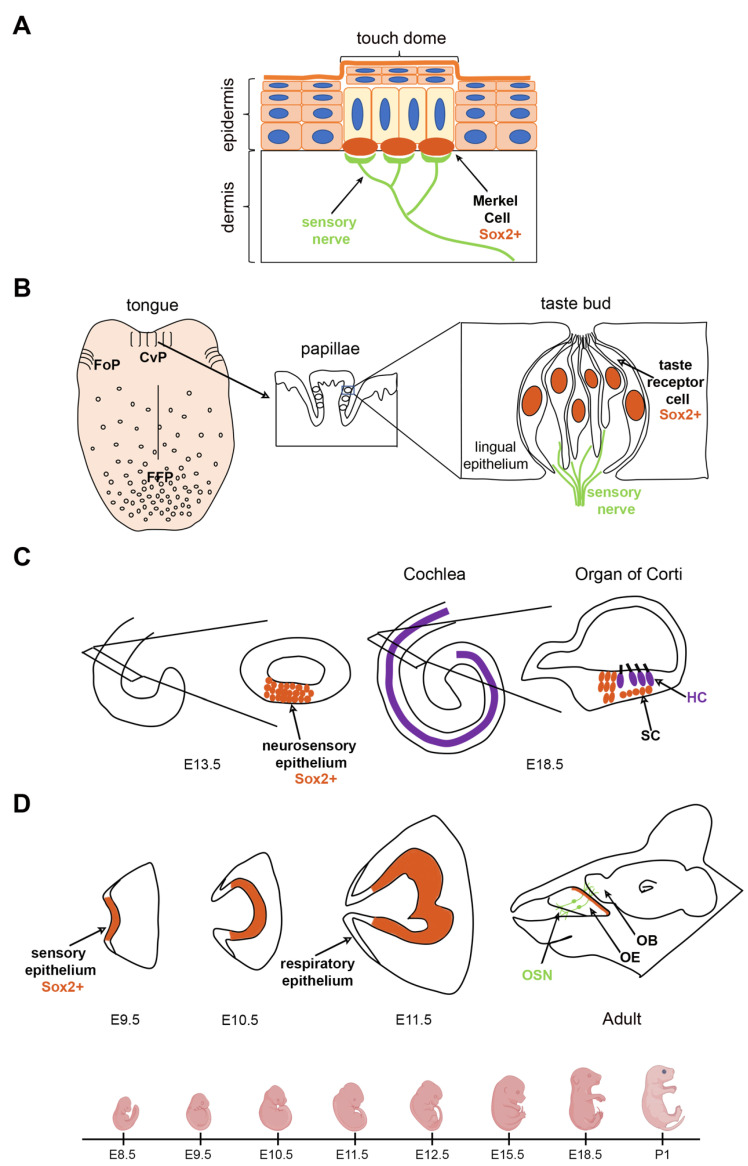
*Sox2* expression during development of sensory organs in mice. (**A**) Schematic representation of a Merkel cell–neurite complex on the skin. *Sox2*-expressing Merkel cells are indicated. (**B**) Schematic representation of the distribution of papillae on the tongue and of the organization of taste buds. Taste receptor cells expressing *Sox2* are indicated. Circumvallate papillae (CvPs), foliate papillae (FoPs) and fungiform papillae (FFPs). (**C**) Schematic representation of inner ear development. *Sox2*-expressing neurosensory epithelium and support cells (SCs) are indicated. Sox-negative hair cells (HCs) are shown. (**D**) Schematic representation of the olfactory system. *Sox2*-expressing sensory epithelium and olfactory epithelium (OE) are indicated. Olfactory sensory neurons (OSNs) projecting to the olfactory bulb (OB) are shown. At the bottom of the figure, a timecourse of mouse development is illustrated.

Mammals have complex tactile organs that sense a variety of touch stimuli; the Merkel cell–neurite complex is one of these organs, and it is responsible for the discrimination of the shape, curvature and texture of objects [18,19,20]. Merkel cell–neurite complexes are formed by Merkel cells and sensory nerve fibers in the skin and are found in touch-sensitive areas such as lips, paw pads, touch domes (specialized mechanosensitive spots in the skin), whisker follicles and finger tips [21] (Figure 1A).

Particularly studied are Merkel cells of whisker follicles that receive somatosensory innervation from axons of the trigeminal ganglion and transmit mechanosensory information to the somatosensory cerebral cortex.

In mice, whisker follicles appear around embryonic (E) day 12. After follicle formation, a Merkel cell–neurite complex composed of Merkel cells and neurites begins to develop starting on E16 and continues until the end of the first postnatal week [22].

Merkel cells express genes of the neuronal lineage such as synaptic vesicle proteins, ion channel subunits and transcription factors important for neural development [23]. They originate from Keratin (Krt) 14-expressing epidermal stem cells in the basal layer of the epidermis [24,25,26,27]. These embryonic epidermal stem cells give rise not only to Merkel cells but also to suprabasal cells, which are involved in forming a barrier to protect the body against the external environment.

### Where Is Sox2 Expressed, and What Does It Do?

*Sox2* expression in Merkel cells is observed starting on E14.5 on mouse skin, and as development proceeds, *Sox2*-expressing Merkel cells increase in number (Figure 1A, Table 1). Differentiating Merkel cells start expressing intermediate filament keratins (Krt) in a sequential manner: initially *Krt8* and then *Krt18* and *Krt20*. In addition, they also express components of presynaptic machinery, such as *Rab3c*. In mature Merkel cells, genes expressed in all differentiation stages are co-expressed. At the end of gestation, *Sox2* is expressed in all *Krt8*-expressing cells [26,28].

To determine the role of SOX2 in Merkel cell formation, the conditional ablation of *Sox2* was generated by crossing a *Sox2*^flox/flox^ line with a *Krt14*-Cre line, in which Cre is expressed in Merkel cells, prior to the onset of *Sox2* expression. *Sox2* was successfully ablated in the back skin and whisker pads of *Sox2*^flox/flox^; *Krt14*-Cre (Sox2-Krt14 cKO) mice. A significant reduction (about 50%) in Merkel cells expressing *Krt8* was observed, and this was not linked to increased apoptosis or reduced proliferation [28]. Interestingly, the remaining *Krt8*-expressing Merkel cells in Sox2-Krt14 cKO mice did not express markers of more differentiated Merkel cells, such as KRT18 or KRT20, indicating that they were still immature [29]. These findings suggest that SOX2 plays a role in regulating Merkel cell differentiation.

A more severe phenotype was obtained when the early Merkel cell marker *Atoh1*, a BHLH transcription factor, was deleted using the same Krt14-Cre line. In this case, no markers of differentiated Merkel cells such as KRT8 or KRT20 were expressed [47]; in addition, no expression of SOX2 was found in *Atoh1* mutant animals [29].

Interestingly, even though *Sox2* and *Atoh1* are expressed at the same time and are the earliest markers of Merkel cells, they have different roles. These data suggest that ATOH1 is important in both Merkel cell specification and the regulation of *Sox2* expression. However, later in development, SOX2 has been shown to be involved in maintaining *Atoh1* expression, which is required for Merkel cell differentiation to proceed.

In fact, in *Sox2* cKO mice, *Atoh1* is expressed in the remaining *Krt8*-expressing Merkel cells [28], but the expression of only *Atoh1* and not *Sox2* is not enough for Merkel cells to complete maturation [24,29]. These findings suggest that ATOH1 is required for Merkel cell specification and that SOX2 is required for their maturation, which involves maintaining *Atoh1* expression [29].

In a search for other transcription factors involved in Merkel cell specification and differentiation, the role of LIM-homeodomain transcription factor ISLET 1 (Isl1) was analyzed. *Isl1* was shown to be specifically expressed in Merkel cells, and not in suprabasal cells, and to be co-expressed with *Atoh1* in both back skin and whisker pads. *Isl1* ablation in Merkel cells using *Krt14*-Cre did not result in Merkel cell anomalies. However, when both *Isl1* and *Sox2* were ablated using *Krt14*-Cre, the phenotype was stronger than in Sox2-Krt14 cKO alone, leading to a greater reduction in *Atoh1* expressing cells [29].

This finding suggests that SOX2 and ISL1 could cooperate to regulate *Atoh1* expression and thus Merkel cell differentiation. Indeed, SOX2 and ISL1 were found to physically interact and to cooperate in activating an enhancer of the *Atoh1* gene [24,29] (Table 1).

In conclusion, ATOH1 is required for Merkel cell specification, while ISL1 and SOX2 function together to sustain *Atoh1* expression in already specified Merkel cells, where ATOH1 is subsequently required for cell differentiation to occur.

## 3. TASTE: SOX2 and Taste Buds

Taste is the sense that we use to choose tasty and healthy foods to eat and avoid toxic ones. Taste buds (TBs) are the end organs of the gustatory system, and they are prevalently localized in three types of papillae on the tongue: anteriorly, in fungiform papillae (FFPs), and posteriorly, in foliate (FoPs) and circumvallate papillae (CvPs). TBs in mice are made up of tens of cells, including taste receptor cells (TRCs), to sense different taste qualities. TB cells have a short life, with half lives of about a week and are continuously renewed by local epithelial stem cells [48]. TBs signal to the brain using the gustatory nerve fibers of the VII and IX cranial nerves.

In mice, initially, primordial placodes of the FFPs appear on the anterior part of the tongue [49]. Subsequently, taste buds (TBs) differentiate as clusters of neuronal cells in the center of the papillae around birth, on postnatal day 0 (P0). Gustatory nerves start to innervate FFPs during gestation, but TB cells are not fully innervated until the end of gestation (E18.5). TBs in FoPs and CvPs form a little later than FFPs and can be seen postnatally (P2–P3) [50] (Figure 1B).

The Shh pathway is very important in TB formation in both embryos and adults, but with different modes of action in these two phases of life. During development, the Shh pathway is required to restrict the regions of the tongue in which placodes will form; in fact, the pharmacological or genetic inhibition of the pathway results in the overproduction of taste buds [51,52]. This effect is probably due to the release of SHH repression in the Wnt pathway, an important positive regulator of TBs during development and regeneration [30,53]. On the other hand, in adults, Shh signaling promotes taste cell differentiation during adult TB renewal [31]. Interestingly, during embryogenesis, TRCs originate from SHH-expressing placodes, while in adults, TRCs are renewed by keratinocytes adjacent to TBs [32].

### 3.1. Where Is Sox2 Expressed, and What Is Its Role?

*Sox2* is expressed at low levels throughout the basal epithelium of the developing and adult mouse tongue and at high levels in TB progenitor cells and in mature TRCs in neonates and adults [31,32] (Figure 1B, Table 1). Lineage-tracing experiments have shown that all TBs in the oral epithelium are derived from *Sox2*-expressing stem cells [54].

*Sox2* deletion in hypomorphic *Sox2* mutants, which express *Sox2* at about 40% of normal levels from the beginning of gestation [9], leads to the complete loss of TBs in the anterior tongue on P0, which is determined with the lack of *Krt8* expression, a marker of mature TB cells [55]. TBs are also lost in other regions of the oral cavity. Interestingly, the loss of TBs does not appear to be due to an increase in apoptosis of developing TBs or to a defect in innervation by gustatory fibers. Therefore, SOX2 is thought to promote, in a dose-dependent way, the formation of TBs versus non-gustatory epithelium/keratinocytes [33].

SOX2 is necessary for TB formation during development, but is it sufficient to induce TBs? *Sox2* overexpression throughout the basal epithelium results in the inhibition of the differentiation of the non-gustatory epithelium and the formation of FFP-like papillae; the latter, however, do not express the TB marker *Krt8* [33]. Therefore, SOX2 inhibits the differentiation of keratinocytes, but cannot induce ectopic TBs by itself [33].

In adult mice, *Sox2* deletion in stem cells of the tongue epithelium in *Sox2*^flox/flox^; *Krt5*-ERT2-Cre (Sox2-Krt5 cKO) mice results in the loss of TBs, and it has been shown to be required downstream of SHH at least in adult taste bud homeostasis [34]. Indeed, SHH overexpression in *Krt14*-expressing progenitors of the adult non-taste epithelium results in elevated SOX2 levels, and these ectopic patches of high SHH/SOX2 expression correspond to ectopic taste buds [35]. These findings suggest that SHH regulates TB homeostasis by increasing SOX2 expression levels. In support of this idea, the inhibition of SHH signaling results in the rapid downregulation of SOX2 in both taste progenitors and TB cells, disrupting taste cell fate [34] (Table 1).

It appears that SOX2 has a more limited function in embryonic lingual epithelium, as opposed to the broader role of SOX2 in the adult tongue [31,34].

Another key pathway involved in TB formation is the Wnt pathway. Wnt signaling is active in the tongue epithelium and promotes TB development and maintenance [30,33]. Indeed, the treatment of tongues from mice expressing GFP under the control of *Sox2* regulatory regions with agonists of the Wnt pathway in early developmental stages (E13.5) results in ectopic GFP expression. These cells are likely still bipotential, with the ability to differentiate in either TBs or keratinocytes.

To confirm the idea that cell types in tongue epithelium expressing high levels of SOX2 can differentiate into TRCs, organoid technologies were used. Cells expressing SOX2 at high levels in adult mouse CvPs are taste-competent progenitors that have the capacity of giving rise to organoids composed of all TRC types. In addition, the ability of *Sox2*-expressing progenitors to give rise to TRCs expressing lingual organoids was increased by the activation of the Wnt pathway, but not the Shh pathway, indicating that SOX2 cooperates with other signaling pathways to induce TRCs [36] (Table 1).

### 3.2. What Are SOX2 Targets Required for Taste Bud Formation?

To identify downstream targets of SOX2 in TB development, genes downregulated in the tongues of *Sox2* hypomorphic mouse mutants were identified. Brain-derived neurotrophic factor (BDNF) and Neurotrophin (Ntf) 3, both expressed in the lingual epithelium, were among the downregulated genes. *BDNF*-null mice were shown to have fewer FFPs than normal, and the double knock-out of *BDNF* and a related Ntf, *Ntf4*, had an even more severe reduction (Mistretta et al. 1999; Nosrat et al. 2004). *Sox2* hypomorphic mutants have a phenotype even more severe than the *BDNF* and *Ntf4* double mutants, suggesting that SOX2 lies upstream of these two factors and potentially of other neurotrophic factors [33] (Table 1).

*Sox2* expression levels vary in the lingual epithelium, and depending on the quantity of SOX2 protein present in lingual progenitors, it is possible to predict if the progenitors will give rise, in vitro, to organoids comprising TRCs or non-taste cells. As mentioned above, cells with high expression of *Sox2* have been shown to be taste-competent progenitors [36]. Among genes enriched in cells with high levels of SOX2 are the transcription factors FOXA1 and FOXA2, which are downstream effectors of the Shh pathway (Table 1). Genes potentially directly regulated by FOXA1/A2 are important mediators of the epidermal–mesenchymal transition (EMT) and include regulators of cell adhesion and cell migration [31]. This suggests that the progenitors of TRCs activate EMT genes to move from the non-taste epithelium into the taste bud environment important in TRC differentiation [56]. Since *Sox2* is highly expressed in these taste-fated progenitors, in the future, it could be interesting to investigate if SOX2 is involved in regulating the EMT.

In conclusion, SOX2 is a key regulator of TB formation and interacts with the Shh and Wnt pathways in these processes. Other signaling pathways, such as the Notch pathway, are also involved in the differentiation of specific TRC types with the regulation of targets genes such as *Hes1* and *Ascl1*, and the involvement of SOX2 in these TRC choices needs further investigation [48]. 

## 4. HEARING: SOX2 and Hair Cells

In mammals, the inner ear is derived from the otic placode, a thickening of the head ectoderm at the level of the hindbrain, in a series of steps. In mice, the otic placode invaginates in the otic cup and forms the otocyst. The otocyst elongates and forms a cochlear pouch and a vestibular pouch [57,58]. Later in development, the vestibular organ is specified, and it includes the utricle, saccule and three semicircular canals. The organ of Corti (oC) develops in the cochlear duct, and when completely differentiated, it is responsible for converting auditory stimuli into neural impulses. The oC is formed by hair cells (HCs) and supporting cells (SCs). HCs in the oC differentiate and form a single row of inner HCs and three rows of outer HCs (Figure 1C, Table 1).

The neurosensory unit of the inner ear is composed of mechano-traducing HCs, supporting cells (SCs) and the primary afferent neurons that connect HCs to neurons in the central nervous system, with the auditory cortex as their final destination. Cochlear HCs are mechanoreceptor cells required for hearing. In the mature oC, IHCs are the primary sensory cells, while OHCs adjust sound perception. Positional variations of HCs establish the tonotopic map consisting of high frequencies at the base and low frequencies in the apical regions. Precise differentiation and patterning are key for correct hearing [59].

### 4.1. Where Is Sox2 Expressed, and What Is Its Role?

*Sox2* is expressed in both neuronal and sensory progenitors and is down-regulated in differentiated neurons and HCs later in development, but it is retained in SCs (Figure 1C, Table 1). Cell-tracing studies have indicated that both HCs and neurons derive from *Sox2*-expressing otic progenitors, suggesting that SOX2 specifies the commitment to the neurosensory state in the early stages of development [37,38]. HCs differentiate from the base to the apex of the cochlea, and *Sox2* expression is higher in undifferentiated precursors than differentiated HCs; indeed, it is downregulated from the base to the apex as development proceeds (Figure 1C).

SOX2 is crucial for neurosensory precursor formation in the otic placode [37,39,40,60]. In fact, mice with reduced or absent SOX2 in the otic epithelium have impaired sensory formation, resulting in reduced or absent HCs and SCs [40]. The characterization of two allelic mouse mutants, light coat and circling (Lcc) and yellow submarine (Ysb), with absence (Lcc) or reduced expression (Ysb) of *Sox2* in the otocyst or adjacent region of the hindbrain, identified hearing and balance impairment. In Lcc, no prosensory domain and no differentiated HCs nor SCs were found. In Ysb/Ysb, abnormal development with disorganized and fewer HCs was observed [40]. To determine if SOX2 is also sufficient to induce HCs, a *Sox2*-expressing construct was electroporated in the chick otic cup or in mouse cochlear explants; these experiments resulted in ectopic HCs, demonstrating that SOX2 can induce HCs when overexpressed in a tissue competent to differentiate into this cell type [41,60]. However, for HC differentiation to occur, at some point, *Sox2* needs to be downregulated, allowing the BHLH transcription factor ATOH1 to be expressed [39,40,42] (Table 1).

### 4.2. What Signaling Pathways Are Upstream of SOX2?

Different signaling pathways have been shown to regulate *Sox2* expression in the otic placode.

A key role has been described for the Shh pathways, which are likely involved in controlling the expression levels of *Sox2* along the basal–apical axis of the otic epithelium [43]. The tight regulation of the levels of the SHH downstream target, GLI2, is required for the proper progression of HC differentiation. It is thought that high expression of *Gli2* delays HC differentiation. In mice in which GLI2 levels are kept high, SOX2 levels are increased, suggesting that GLI2 might inhibit HC differentiation by increasing SOX2 levels. Indeed, SOX2 levels are higher in progenitors and lower in differentiated HCs, and this is controlled by SHH signaling [43].

The expression levels of *Sox2* are critical for HC differentiation in the oC. In fact, the loss of SOX2 leads to the depletion of HCs, while high levels of SOX2 inhibit HC differentiation [39,40,42].

In addition to the Shh pathway, other signaling pathways required for HC differentiation, and potentially HC regeneration, are the Notch and Wnt signaling pathways. Both pathways, independently of each other, control prosensory cell proliferation [39,61,62]. Wnt signaling appears to positively regulate *Atoh1* expression [63,64] and thus promote HC differentiation (Table 1). HCs subsequently express Notch ligands, which, through lateral inhibition, prevent their neighboring cells from becoming HCs; therefore, they become supporting cells (reviewed in [62]).

### 4.3. What Are SOX2 Targets in the Otic Epithelium?

In the neuroepithelium of the inner ear, SOX2 is an upstream regulator of *Atoh1*, which is one of the earliest markers of HC differentiation and is required for their development [40]. In fact, *Atoh1* expression was not present in the inner ear of Lcc/Lcc mice, suggesting that HC differentiation never started in these mice with absent *Sox2* expression. On the other hand, *Sox2* was expressed in *Atoh1* mutants, and the sensory epithelium was specified in these mice, supporting the idea that SOX2 is upstream of *Atoh1*. Other evidence of the SOX2 regulation of *Atoh1* expression is that SOX2 directly binds and activates an *Atoh1* enhancer [37,41,42]. The activation of this enhancer in the mouse cochlea seems to require the cooperation of SOX2 with other transcription factors, which include SIX1, EYA1 and PAX2 [37,41,42] (Table 1).

However, for HCs to differentiate, *Sox2* expression needs to be inhibited so that *Atoh1* expression can increase. Potential *Sox2* inhibitors expressed in the inner ear at the time *Atoh1* starts to be expressed include SOX21. Indeed, in chicks, *Sox21* forced expression when HC formation started inhibited *Sox2* expression, thereby promoting HC differentiation [65].

Another SOX2 target important for HC differentiation is the homeobox transcription factor PROX1, which is expressed in the developing otic sensory epithelium during development and downregulated in HCs at birth. The ectopic expression of *Sox2* in cochlea explants results in ectopic *Prox1* expression; however, cells overexpressing PROX1 do not differentiate into HCs. It is likely that PROX1 mediates the SOX2 antagonistic effects on *Atoh1* expression.

*Prox1* and *Sox2* expression is regulated by the Notch signaling pathway; indeed, the incubation of E13 cochlear explants with the Notch inhibitor DAPT led to inhibition of *Sox2* and *Prox1* expression and thus an increase in HCs [39]. 

In conclusion, SOX2 is required early in development to define neurosensory progenitors in the otic epithelium. It is later required to induce *Atoh1* expression necessary for HC differentiation. *Prox1* expression is induced by SOX2, and it is likely involved in regulating *Atoh1* expression together with SOX2 (Table 1).

## 5. SMELL: SOX2 and Olfactory Sensory Neurons

The olfactory system allows us to process the odorants around us. Sensory neurons are localized in the olfactory epithelium, which originates from the olfactory placode, a thickening of the anterior embryonic head ectoderm. The olfactory epithelium is organized into a sensory domain and a respiratory domain [66,67] (Figure 1D, Table 1). The sensory epithelium produces several cell types, including olfactory sensory neurons, while the respiratory epithelium generates non-neural cells that produce mucus important for the removal of particles from inhaled air.

Olfactory neurogenesis begins in the placodal stage and produces among the first neurons in the vertebrate nervous system [68]. Olfactory neurogenesis requires the activation of specific genes, including *Hes5* in progenitors, *Ngn1* in neural precursors and *NeuroD* in cells committed to leave the cell cycle and become neurons [68,69]. Similar molecular mechanisms control neurogenesis in the embryonic and adult stages in both the olfactory epithelium and the brain [70].

### 5.1. Where Is Sox2 Expressed, and What Is Its Role?

*Sox2* is expressed in the olfactory placode in E9 mice before it invaginates to form the olfactory pit; later, it is expressed in the olfactory epithelium during development and in the adult stages [44,71,72]. The olfactory placode is morphologically visible on E9.5 as an epithelial thickening of the head ectoderm. In this stage, olfactory placodal cells express *Sox2*, and the very first neurons are already present [73]. On E10.5, *Sox2* expression is restricted to the sensory olfactory epithelium and is absent in the respiratory epithelium (Figure 1D, Table 1).

The olfactory epithelium is one of the regions of the CNS, together with the hippocampus and the subventricular zone (SVZ), which has the ability to produce new neurons throughout life [74].

A key role of SOX2 in olfactory system morphogenesis was identified by conditionally ablating *Sox2* only in the forebrain in the early stages of embryogenesis (using Foxg1-Cre [75]) before olfactory placode invagination [45]. In wildtype animals, by E10.5, the olfactory pit was formed, a depression of the head ectoderm in the most anterior–ventral part of the telencephalon, but in Sox2^flox/flox^; Foxg1-Cre cKO (Sox2-Foxg1 cKO) mice, the olfactory pit failed to form, and an extensive cluster of apoptotic cells was observed. A defect in *Sox2* mutants was already present on E9.5 and included reduced proliferation, a slower progression of the cell cycle and the depletion of neurons [45]. Additional evidence of a key role of SOX2 in olfactory neurogenesis comes from the observation that the ectopic overexpression of *Sox2* in the lateral olfactory epithelium enhances neurogenesis and significantly increases the generation of post-mitotic neurons [76].

To determine the cell autonomous involvement of SOX2 in olfactory neurogenesis, avoiding morphological defects, the chick was used as a model organism. *Sox2* expression was inhibited in the chick prospective olfactory region, before olfactory pit formation, by electroporating a vector expressing gRNA against the *Sox2* gene and the hCas9 vector (Sox2-CRISPR-Cas9). As expected, Sox2-CRISPR-electroporated cells did not express *Sox2*, and as in mice, extensive *Sox2* inhibition resulted in a smaller olfactory pit with greatly reduced neurogenesis compared with controls. These results indicate that SOX2’s role in olfactory neurogenesis is conserved in evolution [45].

Recently, it was shown that SOX2 is also required for neuronal production in the olfactory system during regeneration. In the adult olfactory epithelium, *Sox2* is expressed in multipotent and transit-amplifying progenitors, and in an injured olfactory epithelium, it is required to expand the pool of neural progenitors. In fact, if *Sox2* is deleted, olfactory sensory neurons are not regenerated after injury [77].

### 5.2. What Are SOX2 Targets in the Olfactory Epithelium?

The loss of function of SOX2 in the olfactory epithelium of both mice and chicks leads to the downregulation of the neural progenitor gene *Hes5*. In mice and chicks, *Hes5* is the earliest marker associated with neuronal determination in the sensory olfactory epithelium. In *Sox2*-deficient olfactory tissue, the lack of *Hes5*-expressing progenitors leads to the depletion of the whole neuronal lineage, including *Ngn1*-expressing neural progenitors and *NeuroD*-positive neurons, and only a few *Tuj1*-expressing neurons are left. Interestingly, even though *Sox2* is deleted in the whole forebrain in Sox2-Foxg1 cKO mice, *Hes5* is still expressed in the telencephalon, pointing to a context-specific requirement of SOX2 [45] (Table 1).

*Hes5* is a downstream target of Notch signaling [78], but *Notch1* expression is not affected in Sox2-Foxg1 cKO, indicating that the initial expression of *Hes5* in the olfactory epithelium is dependent on SOX2 and not Notch signaling [45]. Indeed, conserved SOX2 binding sites on regulatory regions of the *Hes5* promoter have been identified in different tissues. In neural stem cells in culture, the *Hes5* promoter is activated by increasing the doses of SOX2, and mutations in SOX2 binding sites result in the loss of activation, indicating that SOX2 is a direct regulator of *Hes5* transcription (Table 1). However, *Hes5* expression is not sufficient to induce neurogenesis in the respiratory epithelium, indicating that other genes are required for olfactory sensory neuron specification. *Sox2* deletion leads not only to *Hes5* downregulation, but also to an expansion of the *BMP4* expression domain, usually marking the respiratory (non-neurogenic) epithelium. Therefore, SOX2 promotes the neurogenic lineage in part by restricting *BMP4* expression, thus promoting the sensory domain, and in part by inducing *Hes5* expression. The inhibition of *BMP4* expression is not enough to promote neurogenesis; therefore, there is a direct requirement of SOX2 to promote neurogenesis [45] (Table 1).

In different neural settings, SOX2 requires partners to activate gene transcription [16,17,79]. In the context of the olfactory placode, it was shown that *Sox2*, *Oct1* and *Pax6* are co-expressed in the developing placode and that *Sox2* genetically interacts with *Oct1* to induce the nasal placode. Indeed, *Oct1*^−/−^; *Sox2*^+/−^ mice have no nasal placode, while *Oct1*^−/−^ or *Sox2*^+/−^ have no obvious olfactory defects. In addition, SOX2 and OCT1 together activate the expression of *Pax6* using an ectodermal enhancer, suggesting that this interaction could be required for SOX2-mediated olfactory neurogenesis [46] (Table 1).

In conclusion, SOX2 is required to define the neurogenic domain in the olfactory epithelium by directly activating *Hes5* expression and by limiting *BMP4* expression to the respiratory domain. It likely regulates gene transcription by cooperating with other transcription factors, such as OCT1. Identifying SOX2 targets in the olfactory epithelium could help to identify the gene regulatory network required to induce an olfactory sensory neuron.

## 6. Discussion and Conclusions

In the sensory organs investigated, SOX2 has a key role in the generation, during development, of specific sensory receptor cells. It is expressed in the progenitors of all cell types discussed, which include taste receptor cells, hair cells, Merkel cells and olfactory neurons. In some cases, it needs to be turned off when differentiation proceeds, such as hair cells and olfactory sensory neurons; in other cases, *Sox2* expression is maintained in the differentiated cell types, such as in taste receptor cells and Merkel cells (Table 1). In sensory receptor cells that can regenerate, SOX2 is involved in their regeneration, and its expression is maintained throughout life, such as in the case of taste buds and the progenitors of olfactory sensory neurons [29,38,48,77].

Even though the cell types required to process sensory stimuli are quite different in the different sensory organs and are specialized for very specific functions, SOX2 is important for the development of such different cell types.

Interestingly, the same signaling pathways appear to regulate *Sox2* expression in sensory cells, and some of the transcription factors downstream of SOX2 are also involved in more than one sensory pathway. For example, the Shh pathway is important in inducing *Sox2* expression in taste buds, but also in hair cells [31,42]. Similarly, *Atoh1* is a key target of SOX2 in Merkel cells and in differentiating hair cells [29,39,42] (Table 1).

How can SOX2 regulate the expression of the same gene to direct the differentiation of cell types with completely different functions? On one hand, SOX2 regulates gene expression by associating with different factors, and these factors are different in the different cell types. In fact, SOX2 cooperates with ISL1 to induce *Atoh1* expression in differentiating Merkel cells [24,29], while it cooperates with SIX1, EYA1 and PAX2 to induce *Atoh1* in the cochlea [37,41,42] (Table 1).

On the other hand, SOX2 has recently been shown to be a key factor in regulating long-range interactions in the chromatin in neural stem cells. In particular, SOX2-bound regions are enriched in putative enhancer regions [80,81]. It is possible that SOX2 binds different enhancers of the same gene in different cell types, thereby resulting in the differentiation of such different sensory receptor cells.

It is likely that some SOX2 downstream targets are common to different sensory cell types while others are only specific to one type. Therefore, it is important to determine the gene regulatory network downstream of SOX2 required for the differentiation of the sensory receptor cells discussed in this review, to be able to generate them in vitro or in vivo. Mouse models of *Sox2* deficiency in sensory organs are available (discussed in this review) and genome-wide analyses should be performed, such as RNAseq (using single cells or in bulk), to identify genes deregulated when *Sox2* is deleted that could likely be SOX2 downstream targets. In addition, determining where on the chromatin of the different sensory cell types SOX2 binds could identify direct SOX2 targets and putative regulatory regions.

Innovative culture systems such as organoids could be instrumental to identifying all the key factors required to form a specific sensory organ/sensory cell type, and this system was recently used to investigate the roles of SOX2, SHH and WNT signaling in taste bud formation [36]. Hearing deficits have been described in patients carrying mutations in the *Sox2* gene [12], confirming a key role of SOX2 also in human hair cells and supporting the idea of using SOX2 to regenerate hair cells in vitro or in vivo. However, *Sox2* overexpression alone is often not enough to induce mature sensory receptor cells (for example, hair cells), confirming the need to better understand the gene regulatory network needed for the differentiation of specific sensory receptor cells.

Sensory information from the environment around us, collected with the sensory organs, is transferred to the cerebral cortex, which processes it. The road from the periphery to the final destination is long, and neuronal projection have many relay stations [82]. One of the relay nuclei for visual, somatosensory and acoustic pathways is the thalamus. *Sox2* has been shown to be expressed in the thalamic nuclei that process visual, acoustic and somatosensory stimuli [8]. In these thalamic nuclei, *Sox2* is expressed in differentiated neurons. *Sox2* conditional knock-out in thalamic sensory nuclei disrupts retina–thalamus–cortex connectivity [8]. SOX2 regulates the expression of *Efna5*, a member of the Efna/Eph family of axon guidance molecules, expressed in a gradient in the visual thalamus, the nucleus where visual axons project. The graded expression of *Efna5* is also clearly visible in the thalamic nucleus that receives somatosensory projections; therefore, a characterization of the somatosensory pathway in these *Sox2* thalamic mutants should be carried out [8]. It could be that SOX2 controls the arrival of somatosensory projections in a way similar to visual projections by setting up the graded expression of *Efna5* that guides the arrival of sensory pathways.

## Figures and Tables

**Table 1:** SOX2 and sensory organs in mice.

Sense	Cell Type	*Sox2* Expression Start	Interacting Pathways	SOX2 Targets	SOX2 Co-Factors	Refs.
TOUCH	Merkel cell	E14.5	_	*Atoh1*	ISL1	[24,26,28,29]
TASTE	Taste bud progenitor	E12.5/E13.5	Shh and Wnt	*Ntf3* and *BDNF*	_	[30,31,32,33,34,35,36]
	Taste receptor cell	E18.5/P1				
HEARING	Sensory progenitor	E13.5	Shh and Notch	*Atoh1* and *Prox1*	SIX1, EYA1 AND PAX2	[37,38,39,40,41,42,43]
	Supporting cell	E15.5/E16.5				
	Differentiating hair cells	E15.5				
	Hair cell	Low/not expressed				
SMELL	Olfactory placode	E9	BMP	*Hes5*	OCT1	[44,45,46]
	Sensory epithelium	E10.5				
	Olfactory neuron	Not expressed				

## Data Availability

Data sharing not applicable for this article.
